# Genetic Variants in the Folate Pathway and the Risk of Neural Tube Defects: A Meta-Analysis of the Published Literature

**DOI:** 10.1371/journal.pone.0059570

**Published:** 2013-04-04

**Authors:** Ti Zhang, Jiao Lou, Rong Zhong, Jing Wu, Li Zou, Yu Sun, Xuzai Lu, Li Liu, Xiaoping Miao, Guanglian Xiong

**Affiliations:** 1 Department of Epidemiology and Biostatistics and MOE Key Lab of Environment and Health, School of Public Health, Tongji Medical College, Huazhong University of Science and Technology, Wuhan, China; 2 Department of Epidemiology and Biostatistics, School of Public Health, Guangdong Pharmaceutical University, Guangzhou, China; University of Bonn, Institut of Experimental Hematology and Transfusion Medicine, Germany

## Abstract

**Background:**

Neural Tube Defects (NTDs) are among the most prevalent and most severe congenital malformations worldwide. Polymorphisms in key genes involving the folate pathway have been reported to be associated with the risk of NTDs. However, the results from these published studies are conflicting. We surveyed the literature (1996–2011) and performed a comprehensive meta-analysis to provide empirical evidence on the association.

**Methods and Findings:**

We investigated the effects of 5 genetic variants from 47 study populations, for a total of 85 case-control comparisons *MTHFR* C677T (42 studies; 4374 cases, 7232 controls), *MTHFR* A1298C (22 studies; 2602 cases, 4070 controls), *MTR* A2756G (9 studies; 843 cases, 1006 controls), *MTRR* A66G (8 studies; 703 cases, 1572 controls), and *RFC-1* A80G (4 studies; 1107 cases, 1585 controls). We found a convincing evidence of dominant effects of *MTHFR* C677T (OR 1.23; 95%CI 1.07–1.42) and suggestive evidence of *RFC-1* A80G (OR 1.55; 95%CI 1.24–1.92). However, we found no significant effects of *MTHFR* A1298C, *MTR* A2756G, *MTRR* A66G in risk of NTDs in dominant, recessive or in allelic models.

**Conclusions:**

Our meta-analysis strongly suggested a significant association of the variant *MTHFR* C677T and a suggestive association of *RFC-1* A80G with increased risk of NTDs. However, other variants involved in folate pathway do not demonstrate any evidence for a significant marginal association on susceptibility to NTDs.

## Introduction

Neural Tube Defects (NTDs) are congenital malformations of the brain and spinal cord in neurulation that occur between 21 and 28 days after conception [1]. The most common subtypes of cases include spina bifida, anencephaly and encephalocele. The disease is one of the most prevalent and most severe of birth defects with a high mortality rate [2]. As reported, the average worldwide prevalence is 1 per 1000 living birth [3], in Whites it is approximately the same [4], and in China, it accounts for 20% to 25% of birth defects. Previous research has revealed that the pathogenesis of NTDs is quite complex involving both environmental factors and genetic components.

Folic acid deficiency is relevant to the risk of the disease which was first demonstrated in seminal work done 36 years ago by Smithells et al. which showed that compared with the control group, women who had given birth to NTD children were significantly deficient in several micronutrients, especially folic acid, in diets and postpartum blood [5]. Following observations confirmed that folic acid fortification can prevent the disease to a large extent [6], [7], [8]. Moreover, folic acid supplement, investigated by Berry et al. [9], can prevent NTDs, reducing the incidence by 50–75% without any adverse effects of folic acid for the doses ranging from 0.36 mg (360 µg) to 4 mg (4000 µg) a day. As De-Regil’s described in his review, it can efficiently decrease not only the first occurrence but also the recurrence of the disease among offspring in NTDs families of which parents have had an affected pregnancy [10], [11].

Emerging views of the evidence have begun to shed light on pathogenic mechanisms. One assumption is that folate transport may be affected by immunological responses and maternal autoantibodies that bind to the folate receptor can block the intracellular uptake of folate might cause NTDs [12]. Later studies support that altered folate metabolism contributes to abnormal development of neural system may involve in the etiology of NTDs that reaffirmed the association between the folic acid and the disease. [13], [14].

**Figure 1:**
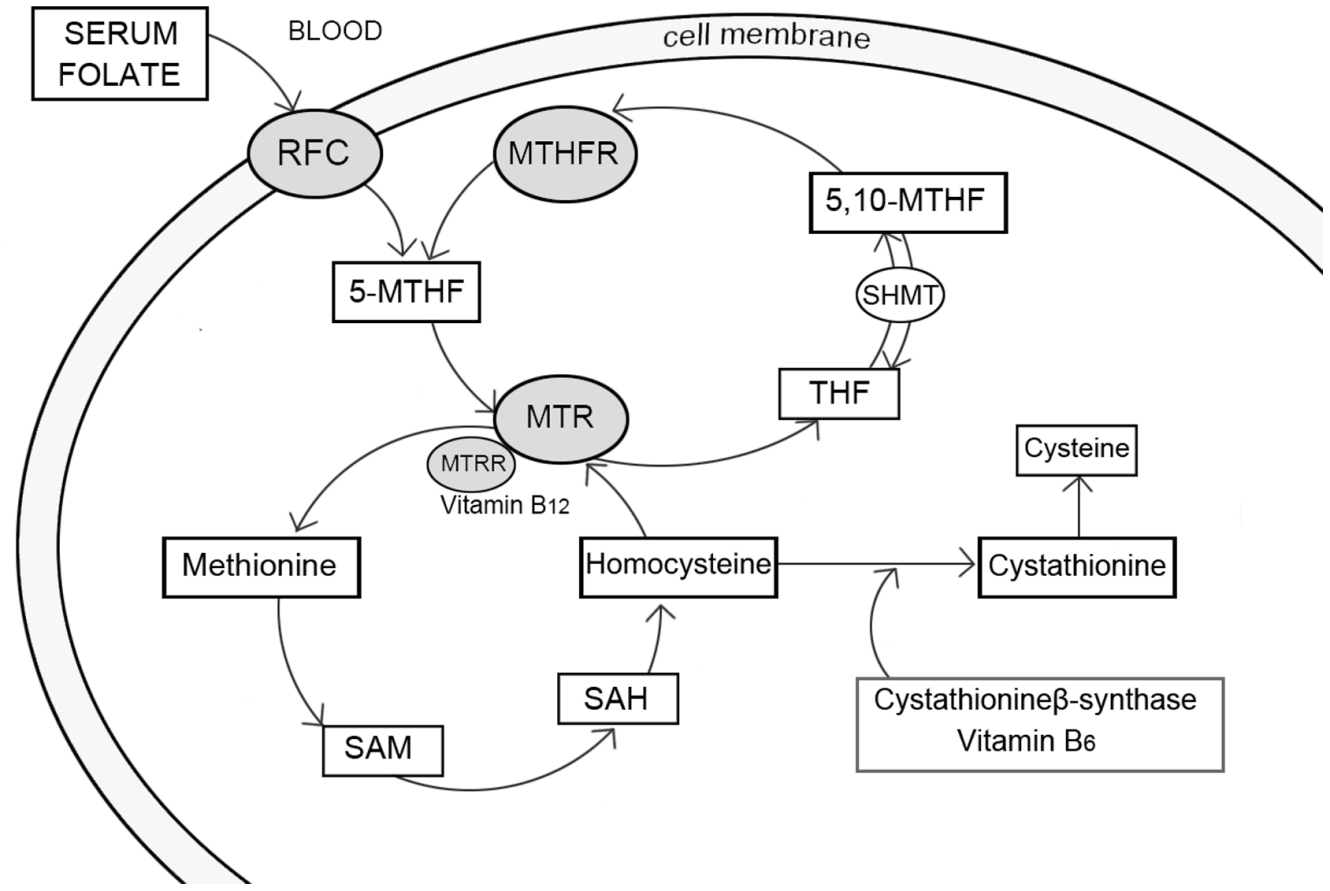
Simplified overview of folate metabolism pathway, highlighting enzymes with polymorphisms investigated in this study. MTHFR, methylene tetrahydrofolate reductase; MTR, methionine synthase; SAH, S-adenosylhomocysteine; SAM, S-adenosylmethionine; MTRR, methionine synthase reductase; THF, tetrahydrofolate. RFC, the reduced folate carrier.

Folic acid must first convert to its naturally bioactive form**–**tetrahydrofolate (THF) and then it can accomplish the methylation cycle in order to function in folate metabolism. Inhibition in the folate metabolism pathway may induce a neural tube defect. Thus, the folate pathway genes that regulate the function of this cycle are widely investigated. Observations showed that some key genes involved in the methylation cycle of THF, such as the methylenetetrahydrofolate reductase (*MTHFR*), the reduced folate carrier (*RFC*) and the methionine synthase reductase (*MTRR*), combine with vitamin B_12_-dependent methionine synthase (*MTR*) function and transfer the methyl group to homocysteine to accomplish the circle [15], [16]. The folate pathway is shown in Figure 1. The association between genetic variance and NTDs was not found until 1995, when the first literature on single-nucleotide polymorphisms (SNPs) appeared [17]. Since then, many articles have shown that the aberrant gene mutations that inhibit cellular folate transportation in folate metabolism have the strongest association with NTDs [10], [18], [19], [20], [21], [22]. SNPs, C677T and A1298C in *MTHFR*, A2756G in MTR, A66G in *MTRR*, A80G in *RFC-1*, have attracted most attention and may represent a substantial proportion of the risk of developing a neural tube defect as their key role in folate metabolism pathway [17]. Increasing evidence from epidemiological case-control studies has revealed that up to 70% in NTD prevalence may result from genetic factors [23].

Interestingly, even though a number of studies investigated the correlation of the NTDs and the polymorphisms, no consensus has been reached. Some observations showed that folate pathway gene polymorphisms might be capable of inhibiting the folate pathway [24], [25], [26], [27], [28]. However, several follow-up studies failed to replicate the association [29], [30], [31], [32]. We conducted this comprehensive meta-analysis integrating previous publications to study the association between key polymorphisms in the major folate pathway genes and NTDs.

## Materials and Methods

To ensure the rigour of this current meta-analysis, we designed and reported it according to the Preferred Reporting Items for Systematic Reviews and Meta-analyses (PRISMA) [33] statement (http://www.prisma-statement.org).

### Search Strategy and Identification of Relevant Studies

We searched PubMed, EMBase, ISI Web of Science, and Chinese Wan Fang Data databases for published articles from June 1996 to May 2011, which investigated at least one of the polymorphisms of *MTHFR, MTRR*, *MTR* and *RFC* associated with NTDs. The search strategy was based on combinations of the English and/or Chinese keywords, “*MTHFR*”, “*MTRR*”, “*MTR*”, “*RFC*”, “folate pathway” “polymorphism”, or “SNP” and “NTDs or Neural Tube Defects or spina bifida” without language restrictions. References of reviews and retrieved studies were also scanned.

The following inclusion criteria had to be fulfilled: (1) case-control study and cohort study design; (2) data on any, some or all polymorphisms in *MTHFR*, *MTRR*, *MTR* and *RFC*; (3) presentation of data necessary for calculating odds ratios (ORs); (4) clear definition of NTDs. Animal studies, mother studies, reviews, and no specific data reported were excluded. Studies that duplicated other studies were eliminated, and only those whose design was complete were finally selected.

### Data Extraction

All the data were extracted independently by two reviewers (T. Zhang & R. Zhong). The following information was extracted from the eligible literature: year of publication, first author’s name, country, ethnicity, genotyping method, source of control, and matching variables of controls with cases. Counts of alleles or genotypes in both case and control groups were extracted or calculated from published data to re-calculate crude ORs and their 95% confidence intervals (95% CIs) for assessing the association of the polymorphisms in *MTHFR*, *MTRR*, *MTR* and *RFC* with NTDs.

### Statistical Analysis

Data from the case-control studies were summarized in two-by-two tables. In each table, crude ORs and their 95% confidence intervals (CIs) were calculated for each individual study based on the genotype data using the method as described by Zintzaras et al. [34]. The Cochran’s *χ* ^2^-based Q statistic test was adopted to assess the case-between heterogeneity and, and heterogeneity was considered significant when *P*<0.05 for Q statistic or when I^2^ was above 75%. Data from the studies were combined in the Mantel-Haenszel Chi-square test by a random-effects model where heterogeneity was significantly present; otherwise, a fixed-effects model was applied. Pooled frequency analysis was performed in the method described by Thakkinstian [35]. Egger’s test and Begg’s test described by Egger et al. [36] for funnel plot asymmetry were applied to evaluate the evidence for publication bias. Models were chosen based on the method described by Thakkinstian [37], briefly, calculating and comparing the ORs of AA vs aa, Aa vs aa and AA and Aa, checking the heterogeneity and significance, then determining the best model. To explore sources of heterogeneity across studies, a meta-regression model was employed [38]. The pre-specified characteristics for assessment of heterogeneity sources were: ethnicity of population (Europe, Native America, Asia, Blacks and Other), source of control (population and hospital based controls), genotyping (PCR-RFLP, PCR-Taqman, Other and NR) and publication year. Stratified analysis, if feasible, was performed in a dominant model based separately on the source of the control group, ethnicity, and genotyping to investigate the reason for heterogeneity. The control group was drawn from three sources: population-based, hospital-based and NR (not reported in literature); by ethnicity, it was divided into 5 groups: European, Native American, Asian, African and Mixed; and by genotyping, it was divided into 4 groups, PCR-RFLP, PCR-Taqman, others and NR. Sensitivity analysis was also conducted to assess the influence of each study on the overall estimate. Cumulative meta-analysis was initially performed by date of publications to determine the dynamic trends as studies accumulated over time. All *P* values are two-tailed with a significance level at 0.05. All statistical analyses were done using Stata Version 10. (College Station, TX: StataCorp LP).

## Results

### Characteristics of Included Studies

**Figure 2:**
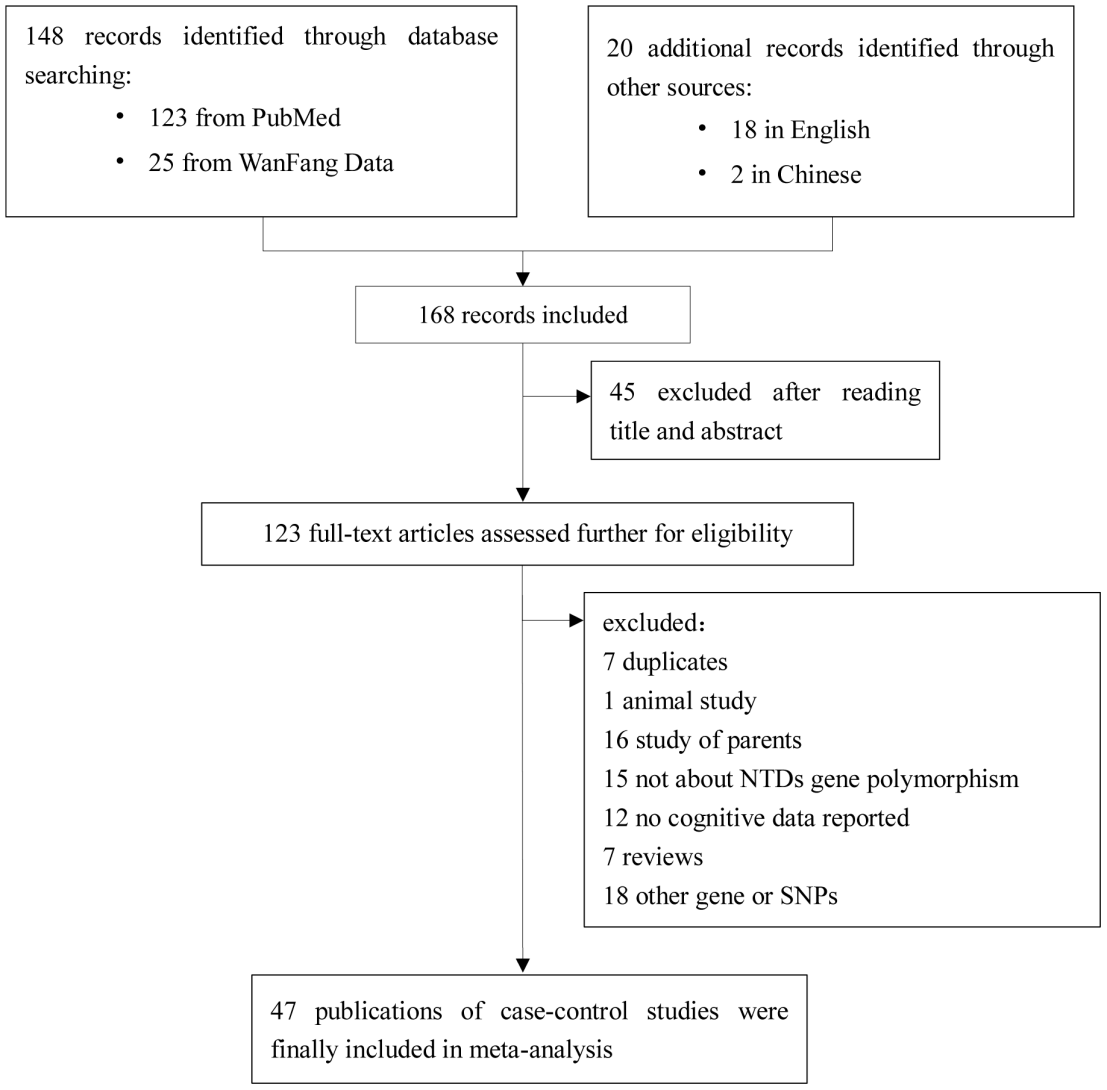
Flow chart of the literature search.

Figure 2 shows the procedure by which literature was selected. A comprehensive search yielded 172 references. After the removal of duplicate literature and articles containing unspecific data that did not meet our criteria, a total of 47 publications was finally included in this meta-analysis. Table S1 illustrates the characteristics of all the literature included in this research. From the table it can be seen that the studies that we investigated consisted of 85 case-control studies, including 42 studies of *MTHFR* C677T, 22 studies of *MTHFR* A1298C, 8 studies of *MTRR* A66G, 9 studies of *MTR* A2756G and 4 studies of A80G. These studies enrolled 4374 cases and 7232 controls of *MTHFR* C677T, 2602 cases and 4070 controls of *MTHFR* A1298C, 703 cases and 1572 controls of *MTRR* A66G, 843 cases and 1006 controls of *MTR* A2756G and 1107 cases and 1585 controls of *RFC-1* A80G. (Table S1).

### Frequency of Risk Allele in the Control Population

**Figure 3:**
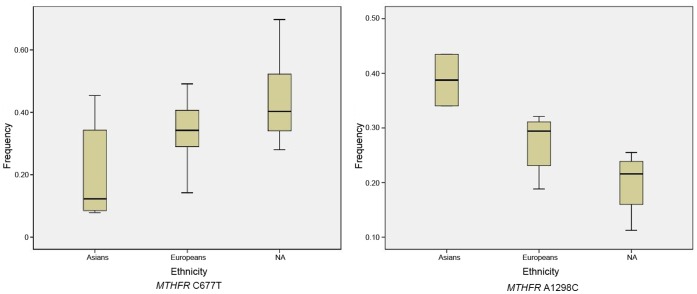
Pooled frequencies of the *MTHFR* C677T alleles and *MTHFR* A1298C alleles in controls stratified by ethnicity. Native A, Native America.

To estimate the pooled frequency, we combined case-control studies of *MTHFR* C677T and A1298C, and extracted data only from the control group. Figure 3 shows the pooled frequency of the variant alleles of *MTHFR* C677T and A1298C that yielded the most publications, in controls stratified by ethnicity. Based on all these samples, the frequency of risk T allele varied among different ethnicities: high in Native American and European healthy populations 43.8% (34.7%–52.9%) and 34.2% (30.7%–37.8%); low in Asian healthy populations 20.7% (11.1%–30.3%). The frequency of risk C allele in A1298C also revealed differences–high in the Asian controls 42.8% (40.3–45.4) and low in Native American and European populations 19.6% (14.4%–24.7%) and 27.2% (24.1%–30.4%) (Figure 3).

### Results of the Overall Meta-analysis

**Figure 4:**
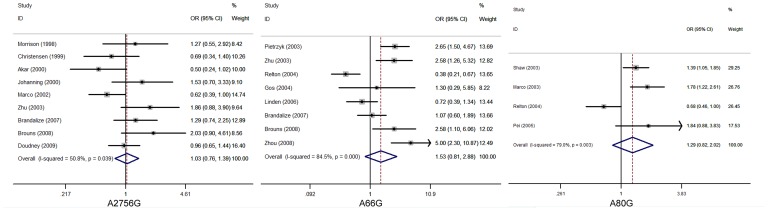
The overall forest plot of OR with 95%CI for *MTR* A2756G, *MTRR* A66G and *RFC-1* A80G polymorphism and Neural tube defects risk in dominant model.

Table 1 summarizes the ORs with corresponding 95% CIs for the association between genetic polymorphisms in the folate metabolism pathway and the risk for NTDs in the dominant, recessive and allelic models. Where significant difference was found in the three genetic models, a random-effects model was chosen according to the *p* values for heterogeneity. A fixed-effects model was applied to the allelic and recessive models in the study of *MTR* A2756G and to the recessive model in the study of *RFC-1* A80G, while a strict random-effects model was chosen for the rest of the studies in which the *p* values of heterogeneity(<0.05) showed significance. According to the method of choosing genetic model, we first calculated the ORs (OR_1_ = 1.335 *p*<0.001, OR_2_ = 1.175 *p* = 0.011, OR_3_ = 1.177 *p* = 0.063), and OR_1_≠OR_2_, so that we excluded the recessive model. Considering that OR_3_ was not significant and the heterogeneity was more significant in an allele model, a dominant model was finally determined. In the same way, we chose a dominant model for the remaining four SNPs. Among all the combined studies of SNPs and NTDs, *MTHFR* C677T showed an association with NTDs (OR 1.23; 95%CI 1.07–1.42). There was no association observed between these four SNPs and NTDs and the results of other SNPs that we performed were negative. Specific ORs, I^2^ and p values are presented below. The results are presented in Figure 4.

**Table 1:** Summarized odds ratios with 95% confidence intervals for the association between genetic polymorphisms in the folate pathway and Neural Tube Defects risk.

Polymorphisms	n^a^	Genetic Model	Model formeta-analysis	OR (95%CI)	*P* forheterogeneity	*I^2^*(%)	*P* for Egger’s test	
*MTHFR* C677T	41	Allele contrast	R	1.18(1.05–1.33)	<0.001	67.4		0.668
	42	Dominant model	R	1.23(1.07–1.42)	<0.001	54.7		0.138
	39	Recessive model	R	1.25(1.03–1.53)	<0.001	52.8		0.653
*MTHFR* A1298C	21	Allele contrast	R	1.05(0.91–1.21)	0.001	55.7		0.802
	22	Dominant model	R	1.09(0.92–1.28)	0.010	46.0		0.867
	21	Recessive model	R	0.96(0.71–1.30)	0.012	45.9		0.437
*MTRR* A66G	8	Allele contrast	R	1.05(0.62–1.78)	<0.001	91.1		0.276
	8	Dominant model	R	1.53(0.81–2.88)	<0.001	84.5		0.587
	8	Recessive model	R	0.80(0.41–1.56)	<0.001	77.6		0.187
*MTR* A2756G	9	Allele contrast	F	0.86(0.71–1.04)	0.118	40.9		0.676
	9	Dominant model	R	1.03(0.76–1.39)	0.039	50.8		0.169
	9	Recessive model	F	0.56(0.31–1.02)	0.357	9.4		0.463
*RFC-1* A80G	4	Allele contrast	R	1.21(0.95–1.56)	0.004	77.4		0.413
	4	Dominant model	R	1.29(0.82–2.02)	0.003	79.0		0.713
	4	Recessive model	F	1.18(0.99–1.39)	0.058	59.8		0.144

a Number of studies. Abbreviation: OR, odds ratio; R, random-effects model; F, fix-effects model.

### Meta-regression Analysis and Stratified Analysis

To explore the potential sources of across study heterogeneity, a meta-regression analysis of *MTHFR* C677T and A1298C was performed respectively. An empty regression was firstly run to estimate the baseline value for τ^2^ (τ_1_^2^ = 0.098 and τ_2_^2^ = 0.244), and then a series univariate model was conducted by adding single covariates including ethnicity of populations, source of controls, publication year and genotyping. In the univariate analysis, only the model including ethnicity and source of controls slightly reduced the τ^2^ value. Then we added the both two covariates, the τ_1_^2^ value reduced to 0.089, R^2^ = 9.47% (*P_1_* = 0.103) and τ_2_^2^ value reduced to 0.196, R^2^ = 22.52% (*P_2_* = 0.196), suggesting ethnicity and source of control cannot explain the major between-study heterogeneity. Studies of *MTHFR* C677T and A1298C were stratified to address heterogeneity while other SNPs were not stratified due to the importance priority and data availability. After stratification by sources of the controls, heterogeneity for *MTHFR* C677T was reduced in the substratification of the hospital based control group. In the population based control group, however, heterogeneity remained and the variant allele still conferred a significant increased risk. After stratification by ethnicity, heterogeneity in the Native American subgroup decreased with *P* = 0.139, I^2^ = 33.6%. Although heterogeneity remained, it should be noted that 22 studies of European subgroups also provided a significant correlation (OR = 1.21; 95%CI 1.03–1.42). For *MTHFR* A1298C, heterogeneity was reduced in the hospital based and Native American subgroups. Nonetheless, no significant association between *MTHFR* A1298C and NTDs was found (Table 2 & 3).

**Table 2:** Stratified analysis of the association between *MTHFR* C677T polymorphism and Neural Tube Defects in dominant model.

MTHFR C677T	n^a^	OR (95% CI)	P forheterogeneity	I^2^(%)
Source of controls				
Population based	19	1.38(1.14–1.68)	0.007	49.2
Hospital based	16	1.10(0.92–1.31)	0.049	39.2
NR	4	1.22(0.59–2.53)	<0.001	80.4
Ethnicity				
Europe	22	1.21(1.03–1.42)	0.004	49.3
Native America	9	1.07(0.81–1.41)	0.139	33.6
Asia	4	1.25(0.62–2.52)	0.002	76.0
Africa	1	1.02(0.40–2.62)	−	−
Mixed	2	2.25(1.05–4.79)	0.015	76.1
Genotyping				
PCR-RFLP	30	1.24(1.06–1.46)	<0.001	52.7
PCR-Taqman	4	1.08(0.66–1.76)	0.028	63.2
Others^b^	4	0.92(0.69–1.24)	0.668	0.0
NR	1	1.77(1.41–2.23)	0.593	0.0

a Number of studies.

b Genotyping including PCR-DHPLC, Dideoxy fingerprinting, Sequenom-based Mass Array assay and Melting Curve Analysis. NR, Not reported.

**Table 3:** Summarized odds ratios with confidence intervals of stratified studies for *MTHFR A1298C* polymorphism.

MTHFR A1298C	n^a^	OR (95% CI)	P forheterogeneity	I^2^(%)
Source of controls				
Populationbased	12	1.07(0.87–1.32)	0.021	51.0
Hospital based	9	1.22(1.02–1.46)	0.332	12.4
NR	1	1.40(0.61–3.21)	−	−
Ethnicity				
Europe	12	1.12(0.75–1.66)	0.015	53.2
Native America	6	0.61(0.30–1.25)	0.818	0.0
Asia	2	1.23(0.30–4.94)	0.056	72.5
Mixed	1	0.44(0.18–1.06)	−	−
Genotyping				
PCR-RFLP	15	0.94(0.66–1.34)	0.023	47.1
PCR-Taqman	2	0.48(0.13–1.73)	0.928	0.0
Others^b^	3	1.02(0.37–2.87)	0.153	46.7
NR	1	3.45(1.00–11.84)	−	−

a Number of studies.

b Other genotyping including PCR-DHPLC, Sequenom-based Mass ARRAY assay and Dideoxy fingerprinting.

### Sensitivity Analysis

We performed a sensitivity meta-analysis to assess the effects of individual studies on pooled ORs. Table 4 shows the results of this sensitivity analysis. None of the studies showed a strong enough influence to affect the combined results in *MTHFR* C677T *and* A1298C, *MTRR* A66G and *MTR* A2756G. After eliminating the results of Marco (2002) [24] in A1298C, heterogeneity decreased (*P* = 0.162, I^2^ = 23.4), which indicated that this study may be the main origin of the heterogeneity. Nevertheless, our results did not change despite removing the data in this study. Relton’s study [27] of A80G affected the association between A80G and NTDs that the results showed a significant correlation with the overall ORs (OR 1.55; 95%CI 1.24–1.92) and no statistical heterogeneity was observed; thus we dropped this study. We will have a more detailed discussion in the following part. The results after the removal were performed in fixed-effects model and identified an overall OR of 1.55(95%CI 1.24–1.92) (Table 4).

**Table 4:** Sensitive analysis of pooled OR for Genetic polymorphisms in MTHFR in the folate pathway.

Study omitted	OR (95% CI)	*P* for heterogeneity	*I^2^*(%)			
For *MTHFR* C677T						
Ou (1996) [1]	1.22(1.06–1.40)	<0.001	54.3			
Mornet (1997) [2]	1.25(1.08–1.43)	<0.001	54.9			
Monsen (1997) [3]	1.22(1.06–1.40)	<0.001	55.1			
Franchis (1998) [4]	1.25(1.08–1.44)	<0.001	54.8			
Morrison (1998) [5]	1.24(1.07–1.43)	<0.001	55.7			
Ubbink (1999) [6]	1.24(1.08–1.42)	<0.001	55.7			
Christensen(1999) [7]	1.23(1.07–1.41)	<0.001	55.7			
Stegmann (1999) [8]	1.23(1.06–1.41)	<0.001	55.4			
Lee (2000) [9]	1.25(1.09–1.43)	<0.001	54.3			
Johanning (2000) [10]	1.20(1.05–1.37)	<0.001	49.3			
Da'valosa (2000) [11]	1.24(1.08–1.43)	<0.001	55.5			
Akar (2000) [12]	1.22(1.06–1.40)	<0.001	54.6			
Volcik (2000) [13]	1.23(1.07–1.42)	<0.001	55.8			
Barber (2000) [14]	1.23(1.07–1.41)	<0.001	55.7			
Fragoso (2002) [15]	1.23(1.07–1.42)	<0.001	55.7			
L (2002) [16]	1.23(1.07–1.42)	<0.001	55.8			
Cunha (2002) [17]	1.25(1.09–1.44)	<0.001	54.0			
McDermott (2003) [18]	1.22(1.06–1.41)	<0.001	54.9			
Perez (2003) [19]	1.22(1.06–1.41)	<0.001	55.4			
Rodriguez (2003) [20]	1.26(1.10–1.44)	<0.001	51.3			
Perez (2003) [19]	1.23(1.07–1.42)	<0.001	55.8			
Rampersaud (2003) [21]	1.26(1.10–1.44)	<0.001	51.2			
Revilla (2003) [22]	1.24(1.08–1.43)	<0.001	55.6			
Pietrzyk (2003) [23]	1.22(1.05–1.40)	<0.001	54.7			
Marco (2003) [24]	1.25(1.09–1.44)	<0.001	53.7			
Volcik (2003) [25]	1.23(1.07–1.42)	<0.001	55.8			
Félix (2004) [26]	1.24(1.07–1.42)	<0.001	55.8			
Relton (2004) [27]	1.24(1.08–1.44)	<0.001	55.1			
Sadewa (2004) [28]	1.24(1.08–1.42)	<0.001	55.3			
Kirke (2004) [29]	1.21(1.06–1.40)	<0.001	51.0			
Gos (2004) [30]	1.24(1.08–1.42)	<0.001	55.7			
Boduroglu (2005) [31]	1.23(1.07–1.42)	<0.001	55.8			
Grandone (2006) [32]	1.23(1.07–1.41)	<0.001	54.6			
Brandalize (2007) [33]	1.24(1.07–1.43)	<0.001	55.7			
Munoz (2007) [34]	1.22(1.06–1.40)	<0.001	54.6			
Zhou (2008) [35]	1.22(1.06–1.40)	<0.001	54.3			
Brouns (2008) [36]	1.23(1.06–1.41)	<0.001	55.5			
Doudney (2009) [37]	1.25(1.09–1.44)	<0.001	53.7			
Behunova (2010) [38]	1.23(1.07–1.42)	<0.001	55.8			
Harisha (2010) [39]	1.21(1.06–1.38)	<0.001	52.4			
Erdogan (2010) [40]	1.24(1.08–1.43)	<0.001	55.6			
Godbole(2011) [41]	1.25(1.08–1.44)	<0.001	53.9			
For *MTHFR* A1298C						
Stegmann (1999) [8]	1.04(0.99–1.10)	0.008	47.7			
Akar (2000) [12]	1.10(0.94–1.30)	0.010	46.6			
Barber (2000) [14]	1.10(0.94–1.30)	0.011	46.4			
Volcik (2000) [13]	1.11(0.94–1.31)	0.012	45.7			
Cunha (2002) [17]	1.09(0.92–1.29)	0.007	48.4			
Marco (2002) [42]	1.04(0.90–1.19)	0.162	23.4			
McDermott (2003) [18]	1.05(0.89–1.24)	0.030	40.2			
Perez (2003) [19]	1.10(0.94–1.30)	0.010	46.6			
Perez (2003) [19]	1.08(0.91–1.28)	0.007	48.4			
Revilla (2003) [22]	1.08(0.91–1.28)	0.008	48.1			
Félix (2004) [26]	1.08(0.91–1.28)	0.007	48.5			
Gos (2004) [30]	1.08(0.91–1.27)	0.008	48.0			
Relton (2004) [27]	1.09(0.91–1.30)	0.007	48.4			
Sadewa (2004) [28]	1.08(0.91–1.27)	0.011	46.4			
Boduroglu (2005) [31]	1.07(0.91–1.27)	0.010	46.9			
Grandone (2006) [32]	1.10(0.93–1.29)	0.009	47.6			
Herrera(2007) [43]	1.09(0.92–1.30)	0.007	48.2			
Munoz (2007) [34]	1.11(0.94–1.31)	0.012	45.7			
Brouns (2008) [36]	1.11(0.94–1.31)	0.011	46.1			
Doudney (2009) [37]	1.08(0.91–1.29)	0.007	48.4			
Behunova (2010) [38]	1.08(0.91–1.28)	0.007	48.2			
Godbole(2011) [41]	1.13(0.96–1.33)	0.051	36.2			
For *MTRR* A66G						
Pietrzyk(2003) [23]	1.40(0.70–2.83)	<0.001	84.5			
Zhu(2003) [44]	1.42(0.70–2.86)	<0.001	85.6			
Relton(2004) [27]	1.90(1.12–3.22)	0.001	72.6			
Gos(2004) [30]	1.55(0.79–3.06)	<0.001	86.7			
Linden(2006) [45]	1.72(0.85–3.48)	<0.001	85.1			
Brandalize(2007) [33]	1.62(0.77–3.43)	<0.001	86.4			
Brouns(2008) [36]	1.42(0.71–2.85)	<0.001	86.0			
Zhou(2008) [35]	1.29(0.69–2.39)	<0.001	81.9			
For *MTR* A2756G						
Morrison(1998) [5]	1.01(0.73–1.41)	0.026	55.9			
Christensen(1999) [7]	1.08(0.78–1.50)	0.033	54.1			
Akar(2000) [12]	1.11(0.82–1.49)	0.083	44.3			
Johanning(2000) [10]	0.99(0.72–1.37)	0.037	53.2			
Marco(2002) [42]	1.12(0.82–1.52)	0.105	41.0			
Zhu(2003) [44]	0.96(0.71–1.31)	0.067	47.1			
Brandalize(2007) [33]	1.00(0.71–1.40)	0.033	54.1			
Brouns(2008) [36]	0.96(0.71–1.30)	0.071	46.4			
Doudney(2009) [37]	1.05(0.73–1.52)	0.023	56.9			
For *RFC-1* A80G						
Shaw(2003) [46]	1.27(0.62–2.60)	0.001	85.3			
Marco(2003) [24]	1.15(0.65–2.03)	0.006	80.6			
Relton(2004) [27]	1.55(1.24–1.92)	0.530	0.0			
Pei(2005) [47]	1.20(0.71–2.02)	0.001	84.9			

### Cumulative Meta-analyses

**Figure 5:**
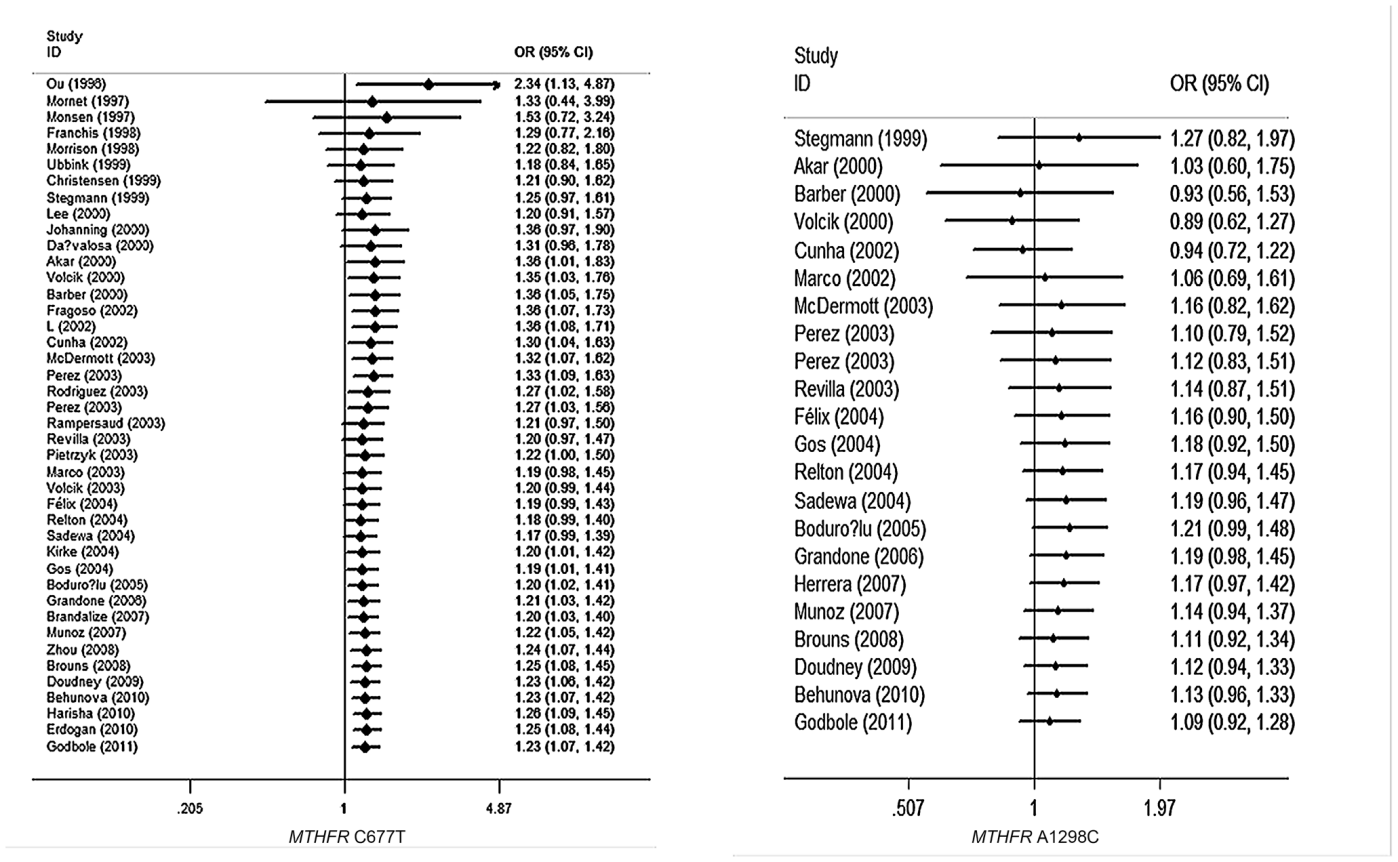
The cumulative forest plot of OR with 95%CI for *MTHFR* C677T polymorphism, *MTHFR* A1298C and Neural tube defects risk in dominant model.

Cumulative meta-analyses were performed using a dominant model for C677T and A1298C, which were the most widely reported in the research. We sorted the literature in chronological order as shown in Figure 3. Remarkably, a statistically significant effect of a positive association between *MTHFR* C677T and NTDs was consistently observed with a narrowing of the 95% confidence interval through publication of the study in 2011. However, association study result of *MTHFR* A1298C and the risk for NTDs was negative. (Figure 5).

### Publication Bias

**Figure 6:**
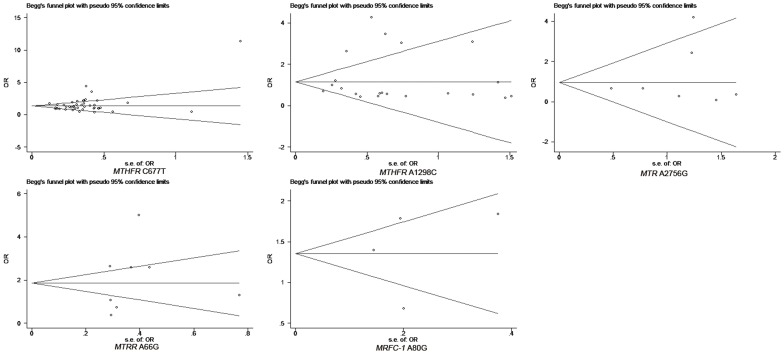
The funnel plot of natural logarithm of OR against inverse standard error in each study.

As demonstrated by the funnel plot and the Egger’s test, there was no significant publication bias in any overall meta analysis. Specific *P*_Egger’s test_ results are presented below. The funnel plots showed in Figure 6.

## Discussion

This current study, to our knowledge, was the first to combine previous studies of key SNPs in the folate metabolism pathway underlying NTDs pathogenesis. Our results demonstrated a significant association between *MTHFR* C677T and NTDs in an overall meta-analysis of case-control studies. Moreover, the association was well supported by the subsequent cumulative meta-analysis. Our overall meta-analysis also integrate studies on A1298C in *MTHFR*, *MTRR* A66G, *MTR* A2756G and *RFC-1* A80G. No significant evidence of correlation btween these SNPs and the NTDs was observed in our study initially. However, after removing one study of *RFC-1* A80G, which was considered the origin of heterogeneity, the results showed a suggestive association.

So far we have known that sufficient folate supplyment during the first four weeks of pregnancy can decrease the risk for NTDs by more than 50% [39] and that folate metabolism and homocysteine status are relevant for the etiology. Mutations of genes in key enzymes in folate metabolism regulate folate transportation and metabolism meanwhile may interfere with its original function, thus leading to birth defects. Studies suggest that several mutations can severely impair *MTHFR* activity, lowering the folate status which could explain a quater of the NTDs occurrence [40], [41]. Fosst et al. [42] first demonstrated that TT substitution at nucleotide(nt) 677 can reduce the *MTHFR* activity by more than 65%, same effect was found in A1298C with less power [43]. This overall meta-analysis indicated that *MTHFR* C677T might be a risk factor of NTDs. Similar results were reported by Motulsky earlier [39] and some follow-up meta-analyses [44], [45]. It’s worth mentioning that the analysis of *MTHFR* C677T included approximately nine times as many participants as N. van der Put’s study (1997) [44] and 9 more studies than M. Amorim (2007) [45], which not only concordant with the ealier two studies but also extended the association to different populations. Although the ORs were not as high as the earliest study reported by Ou [46], with over 4300 cases and 7200 controls, our current analysis would have sufficient statistical power to detect a small size effect in the association between *MTHFR* C677T and NTDs based on current limited knowledge of the exact mechanism.

In addition, the cumulative meta-analysis of *MTHFR* C677T shows a consistantly positive trend with objective facticity. Furthermore, sensitivity analyses have shown that none of the literature we included would influence the results negatively enough to reverse the results.

Nonetheless, the obvious evidence of between-study heterogeneity should be issued in our meta-analysis. The τ^2^ value of meta-regression reduced to 0.089, R^2^ = 9.47% (*P* = 0.103), suggesting ethnicity and source of control cannot explain the major between-study heterogeneity but it may be part of the origin of the heterogeneity. Further, we stratified all the studies into subgroups classified according to source of controls, ethnicity and genotyping. Reduced heterogeneity in Native America subgroup and hospital based subgroup was observed, and significant association was also observed among studies of Europe and Mixed populations. The result of the overall meta-analysis and cumulative meta-analysis for *MTHFR* A1298C did not support the *MTHFR* A1298C as an independent risk factor of NTDs. Considering that C677T and A1298C are both in *MTHFR* and are likely to interact. Also, lack of consensus in the results of individual studies as *MTHFR* C677T and A1298C may be due to the different environment background. The influence of dietary intake, especially folate intake, varied in different ethnic populations, which may well be relevant to the difference in prevalence and cannot be excluded in the this study. The between-study heterogeneity would also affect the results. However, the meta-regression analysis suggested that ethnicity and source of control may not be the major origin of the heterogeneity. In stratified analysis, we observed the heterogeneity reduced sharply only in Native American group and Taqman group. After checking all possible sources of errors, with the avalaible data we failed to exclude the influence of the between-study heterogeneity that existed in a relatively large meta-analysis. The heterogeneity might be due to many reasons, such as differences in maternal folate status and recruitment procedures of the study population.

Under the hypothesis that loss-of-function mutations in *MTR* and mutations of the chaperone *MTRR* related to the activity of *MTR* may influence homocysteine levels resulting in severe disease phenotypes [47] [48], we combined the data of the key variants in *MTHFR*, *MTRR* and *MTR* that are in folate metabolisms to analyse the association. However, the available evidence did not support *MTRR* A66G or *MTR* A2756G as an independent risk factor of NTDs. Some explanation might be responsible for the lack of correlation. First, the sample size of studies was relatively small, so that to detect a very small size effect may require much larger sample size. Second, the outcome of an NTD patient varies from livebirth to stillbirth as the severity varies in different subtypes. Thus, the effect of genetic variants on risk of NTDs may be underestimated if studies only collect livebirths and less severity cases. Third, potential gene-gene, gene-environment interaction may affect the current results.

The sensitivity analysis of *RFC-1* A80G showed that the study of Relton (2004) [29] affected the results which should be figured out. Under review of this report, Relton et al. indicated a contradictory result to other included studies that the 80A allele, not the 80G allele, increased risk of NTDs. Then we excluded the possibility that they report the allele for the reverse strand. Additionally, after removing this study, the heterogeneity reduced sharply, which revealed it was the main origin of the heterogeneity. The result of *RFC-1* A80G in fixed-effects model was 1.55(95%CI 1.24–1.92). Since the sample size was quite limited (901 cases and 983 controls), we must be cautious of the association and more studies should be required to add in to improve the precision of the result.

The current study strongly supports the association of *MTHFR* C677T alleles with NTD risk by performing a cumulative meta-analysis of 42 studies of *MTHFR* C677T that demonstrated results with a relatively narrow 95% confidence interval. We believe that as more studies are added to our meta-analysis, results would remain stable. Also, we observed a suggestive association beween RFC-1 A80G and the risk of NTDs. However we failed to find a correlation in the remaining SNPs.

Some limitations merit serious consideration in our meta analysis. Firstly, as the pressent meta-analysis was primarily based on unadjusted effect estimates, the confounding factors were not controlled. Additionally, with the eligible information and methodological limitations we cannot excluded the between-study heterogeneity that remained. Secondly, the effects of gene-gene and gene-environment interaction was not addressed in this study. Thirdly, we systematically searched a variaty of databases for published literature, however, we cannot excluded the possiblity of missing some.

In summary, our studies demonstrated a significant correlation of *MTHFR* C677T, and a suggestive association of *RFC-1* A80G and the increased risk of NTDs while the other SNPs in our study failed to support an evidence of the association. As the low edge of 95%CI nearly touched the null value, our research should be viewed with caution. Further large and well-designed studies will be needed to clarify the association of the polymorphisms in the folate pathway genes.

## Supporting Information

Table S1 **Characteristics of studies on genetic polymorphisms in the folate pathway and Neural Tube Defects risk included in the meta-analysis.**
(DOC)

Checklist S1 **PRISMA Checklist.**
(DOC)
