# Supplementary material for: Genetic Variants in the Folate Pathway and the Risk of Neural Tube Defects: A Meta-Analysis of the Published Literature
Source: PLoS One. 2013 Apr 4;8(4):e59570. doi: 10.1371/journal.pone.0059570 (PMC3617174; doi:10.1371/journal.pone.0059570)
Supplement: Table S1 — Characteristics of studies on genetic polymorphisms in the folate pathway and Neural Tube Defects risk included in the meta-analysis. (DOC) [file pone.0059570.s001.doc]

**Table S1. Characteristics of studies on genetic polymorphisms in the folate pathway and Neural Tube Defects risk included in the meta-analysis.**

| **First author** | **Year of publication** | | | **Country (region)** | | **Ethnicity** | | **Control source** | | **Sample size (cases/ controls)** | **Genotyping**  **Method** | **HWE** | **Matching criteria** |
| --- | --- | --- | --- | --- | --- | --- | --- | --- | --- | --- | --- | --- | --- |
| ***MTHFR* C677T** | | | | | | | | | | | | | |
| Ou[1] | 1996 | | | USA | | Mixed | | PB | 41/109 | | TaqMan | Y | NR |
| Mornet[2] | 1997 | | | France | | Europe | | HB | 43/133 | | RFLP | Y | ethnicity |
| Monsen[3] | 1997 | | | Norway | | Europe | | PB | 28/68 | | RFLP | Y | ethnicity, age |
| Franchis[4] | 1998 | | | Italy | | Europe | | HB | 203/583 | | RFLP | N | NR |
| Morrison[5] | 1998 | | | UK | | Europe | | NR | 51/199 | | TaqMan | Y | ethnicity |
| Ubbink[6] | 1999 | | | South Africa | | Africa | | HB | 53/54 | | RFLP | Y | age, body mass, ethnicity |
| Christensen[7] | 1999 | | | Canada | | Europe | | PB | 56/97 | | RFLP | Y | NR |
| Stegmann[8] | 1999 | | | Germany | | Europe | | PB | 148/174 | | RFLP | Y | ethnicity |
| Lee[9] | 2000 | | | South Korea | | Asia | | HB | 21/43 | | RFLP | Y | age, ethnicity, region |
| Johanning[10] | 2000 | | | USA | | Mixed | | NR | 82/76 | | RFLP | Y | NR |
| Da´valosa[11] | 2000 | | | Mexico | | Native America | | PB | 107/101 | | RFLP | Y | NR |
| Akar[12] | 2000 | | | Turkey | | Europe | | PB | 56/76 | | NR | Y | region, ethnicity |
| Volcik[13] | 2000 | | | USA | | Europe | | PB | 234/112 | | TaqMan | Y | ethnicity, region |
| Barber[14] | 2000 | | | USA | | Native America | | PB | 24/93 | | Dideoxy fingerprinting | N | Region |
| Fragoso[15] | 2002 | | | Puerto Rico | | Native America | | HB | 31/100 | | RFLP | Y | region, ethnicity |
| L[16] | 2002 | | | Mexico | | Native America | | NR | 65/110 | | RFLP | Y | region |
| Cunha[17] | 2002 | | | Brazil | | Native America | | PB | 100/100 | | RFLP | Y | region |
| McDermott[18] | 2003 | | | Ireland | | Europe | | HB | 276/255 | | RFLP | Y | NR |
| Perez[19] | 2003 | | | Brazil | | Europe | | HB | 81/51 | | RFLP | Y | age, ethnicity |
| Rodriguez[20] | 2003 | | | Italy | | Europe | | HB | 40/58 | | RFLP | Y | region, ethnicity |
| Perez[19] | 2003 | | | Brazil | | Native America | | HB | 50/75 | | RFLP | Y | age, ethnicity |
| Rampersaud[21] | 2003 | | | USA | | Native America | | NR | 175/195 | | TaqMan | Y | ethnicity |
| Revilla[22] | 2003 | | | Spain | | Europe | | NR | 27/159 | | RFLP | Y | NR |
| Pietrzyk[23] | 2003 | | | Poland | | Europe | | PB | 104/100 | | RFLP | Y | ethnicity |
| Marco[24] | 2003 | | | Italy | | Europe | | PB | 168/138 | | RFLP | Y | age, sex, ethnicity |
| Volcik[25] | 2003 | | | USA | | Native America | | PB | 100/100 | | RFLP | Y | NR |
| Félix[26] | 2004 | | | Brazil | | Native America | | HB | 41/44 | | TaqMan | Y | region, ethnicity |
| Relton[27] | 2004 | | | UK | | Europe | | PB | 200/578 | | RFLP | Y | region, ethnicity |
| Sadewa[28] | 2004 | | | Indonesia | | Asia | | PB | 13/47 | | PCR-DHPLC | Y | region,ethnicity |
| Kirke[29] | 2004 | | | Ireland | | Europe | | PB | 395/848 | | NR | Y | ethnicity |
| Gos[30] | 2004 | | | Poland | | Europe | | PB | 20/262 | | RFLP | Y | ethnicity |
| Boduroğlu[31] | 2005 | | | Turkey | | Europe | | HB | 190/186 | | RFLP | Y | age |
| Grandone[32] | 2006 | | | Italy | | Europe | | PB | 15/143 | | RFLP | Y | ethnicity |
| Brandalize[33] | 2007 | | | South Brazil | | Europe | | HB | 114/100 | | RFLP | Y | region, ethnicity |
| Munoz[34] | 2007 | | | Mexico | | Native America | | PB | 118/112 | | RFLP | Y | age, intensity. Region |
| Zhou[35] | 2008 | | | China | | Asia | | HB | 71/140 | | RFLP | Y | age, sex, ethnicity |
| Brouns[36] | 2008 | | | The Netherlands | | Europe | | PB | 71/102 | | RFLP | NR | ethnicity |
| Doudney[37] | 2009 | | | UK | | Europe | | HB | 287/187 | | RFLP | Y | ethnicity |
| Behunova[38] | 2010 | | | Slovakia | | Mixed | | HB | 92/290 | | RFLP | Y | NR |
| Harisha[39] | 2010 | | | India | | Asia | | PB | 45/102 | | RFLP | Y | intensity, ethnicity |
| Erdogan[40] | 2010 | | | Turkey | | Europe | | PB | 33/48 | | Melting Curve Analysis | Y | region |
| Godbole[41] | 2011 | | | India | | Asia | | HB | 305/684 | | Sequenom-based Mass ARRAY Assay | Y | mother's age, sex, weight, ethnicity, history, type |
| ***MTHFR* A1298C** | | | | | | | | | | | | | |
| Stegmann[8] | | 1999 | Germany | | | | Europe | PB | 148/174 | | RFLP | Y | ethnicity |
| Akar[12] | | 2000 | Turkey | | | | Europe | PB | 56/76 | | NR | Y | region, ethnicity |
| Barber[14] | | 2000 | USA | | | | Native America | PB | 19/85 | | Dideoxy ﬁngerprinting | Y | region |
| Volcik[13] | | 2000 | USA | | | | Europe | PB | 233/82 | | TaqMan | Y | ethnicity, region |
| Cunha[17] | | 2002 | Brazil | | | | Native America | PB | 100/100 | | RFLP | Y | region |
| Marco[42] | | 2002 | Italy | | | | Europe | PB | 203/202 | | RFLP | Y | age, sex, ethnicity |
| McDermott[18] | | 2003 | Ireland | | | | Europe | HB | 276/354 | | RFLP | Y | NR |
| Perez[19] | | 2003 | Brazil | | | | Europe | HB | 81/51 | | RFLP | Y | age, ethnicity |
| Perez[19] | | 2003 | Brazil | | | | Native America | HB | 50/75 | | RFLP | Y | age, ethnicity |
| Revilla[22] | | 2003 | Spain | | | | Europe | NR | 27/159 | | RFLP | Y | NR |
| Félix[26] | | 2004 | Brazil | | | | Native America | HB | 41/44 | | TaqMan | Y | region, ethnicity |
| Gos[30] | | 2004 | Poland | | | | Europe | PB | 20/262 | | RFLP | Y | ethnicity |
| Relton[27] | | 2004 | UK | | | | Europe | PB | 194/584 | | RFLP | Y | region, ethnicity |
| Sadewa[28] | | 2004 | IndoNsia | | | | Asia | PB | 13/47 | | PCR-DHPLC | Y | region,ethnicity |
| Boduroğlu[31] | | 2005 | Turkey | | | | Europe | HB | 190/186 | | RFLP | N | age |
| Grandone[32] | | 2006 | Italy | | | | Europe | HB | 15/143 | | RFLP | Y | ethnicity |
| Herrera[43] | | 2007 | Mexico | | | | Native America | PB | 108/120 | | RFLP | Y | ethnicity |
| Munoz[34] | | 2007 | Mexico | | | | Native America | PB | 92/80 | | RFLP | Y | age, intensity, region |
| Brouns[36] | | 2008 | Ntherlands | | | | Europe | HB | 71/105 | | RFLP | NR | ethnicity |
| Doudney[37] | | 2009 | UK | | | | Europe | HB | 273/176 | | RFLP | Y | ethnicity |
| Behunova[38] | | 2010 | Slovakia | | | | Mixed | HB | 92/290 | | RFLP | Y | NR |
| Godbole[41] | | 2011 | India | | | | Asia | HB | 300/675 | | Sequenom-based Mass ARRAY Assay | Y | mother's age, sex, weight, ethnicity, history, type |
| ***MTRR* A66G** | | | | | | | | | | | | | |
| Pietrzyk[23] | | 2003 | Poland | | | | Europe | PB | 104/100 | | RFLP | Y | ethnicity |
| Zhu[44] | | 2003 | USA | | | | Europe | PB | 43/124 | | RFLP | NR | ethnicity, region |
| Relton[27] | | 2004 | UK | | | | Europe | PB | 201/601 | | RFLP | N | region, ethnicity |
| Gos[30] | | 2004 | Poland | | | | Europe | PB | 20/262 | | RFLP | N | ethnicity |
| Linden[45] | | 2006 | Ntherlands | | | | Europe | HB | 99/213 | | RFLP | Y | NR |
| Brandalize[33] | | 2007 | South Brazil | | | | Europe | HB | 114/100 | | RFLP | N | region, ethnicity |
| Brouns[36] | | 2008 | Ntherlands | | | | Europe | HB | 58/68 | | RFLP | NR | NR |
| Zhou[35] | | 2008 | China | | | | Asia | HB | 64/104 | | RFLP | Y | age, sex, ethnicity |
| ***MS* A2756G** | | | | | | | | | | | | | |
| Morrison[5] | | 1998 | UK | | | | Europe | NR | 36/72 | | TaqMan | Y | ethnicity |
| Christensen[7] | | 1999 | Canada | | | | Europe | PB | 55/97 | | RFLP | Y | NR |
| Akar[12] | | 2000 | Turkey | | | | Europe | PB | 56/76 | | NR | Y | region, ethnicity |
| Johanning[10] | | 2000 | USA | | | | Mixed | NR | 77/84 | | RFLP | Y | NR |
| Marco[42] | | 2002 | Italy | | | | Europe | PB | 174/210 | | RFLP | N | NR |
| Zhu[44] | | 2003 | USA | | | | Europe | PB | 43/124 | | RFLP | NR | ethnicity, region |
| Brandalize[33] | | 2007 | South Brazil | | | | Europe | HB | 114/100 | | RFLP | Y | region, ethnicity |
| Brouns[36] | | 2008 | The Netherlands | | | | Europe | HB | 57/55 | | RFLP | NR | ethnicity |
| Doudney[37] | | 2009 | UK | | | | Europe | HB | 231/188 | | RFLP | Y | ethnicity |
| ***RFC-1* A80G** | | | | | | | | | | | | | |
| Shaw[46] | | 2003 | USA | | Mixed | | | PB | 468/603 | | RFLP | N | age, region |
| Marco[24] | | 2003 | Italy | | Europe | | | PB | 329/281 | | RFLP | Y | age, sex, ethnicity |
| Relton[27] | | 2004 | UK | | Europe | | | PB | 206/602 | | RFLP | Y | region, ethnicity |
| Pei[47] | | 2005 | China | | Asia | | | PB | 104/99 | | RFLP | Y | age, region, ethnicity |

Abbreviations: HWE, Hardy–Weinberg equilibrium in control group; PB, Population-based study; HB, hospital-based study; NR, not reported; Y yes, N no; RFLP, Restriction Fragment Length Polymorphism; PCR-DHPLC, Polymerase Chain Reaction- Denatured High Performance Liquid Chromatography.
